# Taking a critical look at the UNAIDS global estimates on paediatric and adolescent HIV survival and death

**DOI:** 10.7448/IAS.20.1.21952

**Published:** 2017-06-28

**Authors:** IeDEA Pediatric Working Group

**Keywords:** HIV, pediatric, children, adolescents, global, estimates, modeling, Spectrum

In 2016, UNAIDS revised their global estimates of children and adolescents becoming infected with, living, and dying with HIV [1]. The result was to reduce the total number of children under 15 years living with HIV in 2015 by 800,000 - down to 1.8 million - and of adolescents 10–19 years by 200,000, down to 1.8 million (aidsinfo.unaids.org). In addition, a reanalysis of 2012 estimates presented at the AIDS 2016 conference led to a correction of an earlier report by WHO [2] which moved the ranking of HIV/AIDS from the second leading cause of adolescent death down to eighth place, due to a dramatic decrease in the estimated number of deaths from 98,500 to 43,400 [3]. However, these changes were largely not due to actual improvements in HIV diagnosis or starting children and adolescents on life-saving antiretroviral therapy (ART) sooner. Instead, they were the functional result of changes made to the UNAIDS Spectrum analytical model that has been the primary method of estimating the size of global and regional HIV epidemics [4–6]. Global paediatric HIV estimates require simulation modelling to calculate the denominators of children living with HIV, because national programme data are not consistently robust enough to represent true numbers of those infected, treated or dying from the disease, and such data do not exist for infants and children who never enrol in HIV programmes [6,7].

The Spectrum team regularly updates its model to refine global HIV estimates. For 2016, the Spectrum team substantially revised the structure of the paediatric HIV model, reflecting key differences between paediatric and adult HIV. This included two major updates to the data used as model inputs, which directly impacted the 2016 estimates. First, new data suggested lower rates of mother-to-child HIV transmission for different categories of women at risk (e.g., from 30% to 18% if seroconverting during pregnancy). This resulted in fewer infants estimated by Spectrum to be infected at birth. Second, Spectrum incorporated newly available age-disaggregated data into the Spectrum model on when children started antiretroviral therapy, provided by the International Epidemiology Databases to Evaluate AIDS global consortium (IeDEA.org), an observational cohort including >140,000 children and adolescents in low and middle-income countries (91% from sub-Saharan Africa) [8]. The IeDEA data showed that a large proportion of young children were not starting treatment at the ages recommended by WHO or country guidelines, as had been assumed in the previous UNAIDS model. As a result, the estimated mortality in infected children aged <10 years increased, because Spectrum no longer assumed they were diagnosed and treated soon enough to prevent early HIV-associated mortality [9,10]. While the uncertainty bounds of the earlier estimates of new infections and of deaths among infected children overlapped with the revised estimates in the updated model, these two changes left fewer perinatally infected children and adolescents surviving in Spectrum to be included in the estimates of youth living with HIV [1].

Another important effect of the Spectrum revisions was that estimated global ART coverage for children <15 years rose from 30% to 50%. This occurred to a small degree because more children were reported by HIV programmes to be treated with ART, but to a larger degree because the number of children living with HIV - the denominator in ART coverage - was lower. Consequently, the apparent improvements in ART coverage should not necessarily be interpreted as progress in the global HIV response, but are a reminder that model input parameters are based on assumptions that rely on limited actual paediatric and age-disaggregated data.

The magnitude of the changes in the 2016 Spectrum estimates have had the effect of focusing the attention of the HIV community on the processes and assumptions that led to both previous and current model outputs. Prior to 2016, Spectrum mortality assumptions for untreated HIV-infected infants were based on a pooled analysis of empiric data in young children [11]; for children aged >2.5 years, mortality rates from young adults infected between 15 and 24 years of age were applied [11]. In addition, modelled mortality for children on ART was based on an earlier analysis of IeDEA data [7]. The 2016 Spectrum revisions built on the strength of the IeDEA cohort’s size and geographic coverage; however, the IeDEA database is a curated research database which may have limitations in terms of representativeness relative to national data and utility in monitoring country-level progress on paediatric HIV.

Well into the second decade of the worldwide ART roll-out, the lack of high-quality data from a broad range of HIV care and treatment programmes is of great concern. This is particularly important given the magnitude of the resource-allocation and policy decisions which are based on the Spectrum estimates. Investing in improved patient- and program-level data is critical to inform our progress towards various benchmarks, such as reaching the UN’s “90-90-90” goals [12]. These progress measures also assist with forecasting antiretroviral drug production needs for infant and child formulations, guiding the allocation of financial, human, and technical resources by HIV donor agencies, and setting research priorities. A key user of Spectrum estimates is the US PEPFAR programme, whose country operating budgets totalled $3.76 billion in 2015, and $36.15 billion since 2005 (copsdata.amfar.org), and is an entity for which the availability of data for programme reporting purposes is mandated by US law [13].

Although new UNAIDS estimates are accompanied by carefully articulated caveats and include detailed descriptions of the levels of uncertainty, public policy decisions have been based on the point estimates themselves. While Spectrum estimates remain essential benchmarks, readers should understand their limitations in the context of children and youth and be aware of the assumptions that have led to the changes in paediatric HIV estimates over time. Beyond that, we need a renewed emphasis on supporting the infrastructure required for data collection, management, and analysis within national HIV programmes in low-income and middle-income countries. In addition, there is a need for model validation, in which Spectrum estimates are compared to empiric data for outcomes such as perinatal HIV transmission and ART coverage. Investments in higher quality data, transparency in analytical approaches and assumptions underpinning mathematical modelling methods, and data-sharing will lead to more reliable estimates. These can better drive the research agenda around programme implementation and quality improvement throughout the treatment cascade and across a child’s transitions into adult life, facilitating our assessments of progress towards the 90-90-90 targets for children and adolescents and optimizing our ability to control the epidemic.
